# Association of Crohn’s disease with periodontal disease risk and severity: a meta-analytic study

**DOI:** 10.2340/aos.v85.45706

**Published:** 2026-04-30

**Authors:** Yanyan Jin, Songhai Duan, Weidong Chu, Weijia Shen

**Affiliations:** Department of Stomatology, Haining People’s Hospital, Jiaxing City, China

**Keywords:** Crohn’s disease, periodontitis, gingivitis, inflammatory bowel disease, meta-analysis, systematic review

## Abstract

**Background:**

Crohn’s disease (CD) is a chronic inflammatory disorder with systemic manifestations, including potential effects on oral health. Evidence regarding its association with periodontal disease remains inconsistent.

**Methods:**

We systematically searched PubMed, Embase, Web of Science, Scopus, Cochrane Library, CNKI, and WanFang Data up to August 28, 2025, for observational studies comparing periodontal outcomes in adults with CD and non-inflammatory bowel diseases controls. Random-effects meta-analyses were performed to estimate pooled odds ratios (ORs) for prevalence and standardized mean differences (SMDs) for clinical parameters. Subgroup and sensitivity analyses were conducted.

**Results:**

Thirty-four studies, including 6482 CD patients and 9137 controls, were analyzed. CD was associated with a higher risk of periodontitis (OR = 2.14, 95% confidence interval [CI]: 1.65–2.77). Clinical periodontal parameters were also significantly worse in CD patients: probing depth (SMD = 0.42), clinical attachment loss (CAL) (SMD = 0.50), bleeding on probing (SMD = 0.47), plaque index (SMD = 0.39), and gingival index (SMD = 0.31). Associations were stronger in European populations and in studies using CAL criteria. Sensitivity analyses confirmed the robustness of results.

**Conclusions:**

CD is associated with increased prevalence and severity of periodontal disease. These findings support routine periodontal screening and integrated multidisciplinary management for CD patients, highlighting shared inflammatory pathways as potential therapeutic targets.

## GRAPHICAL ABSTRACT

**Figure:**
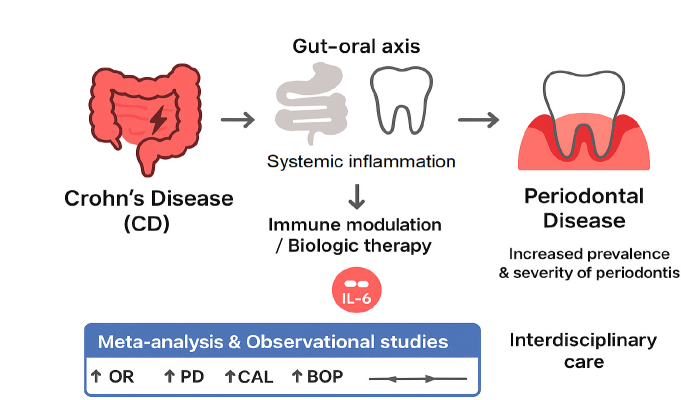

## Introduction

Crohn’s disease (CD) is a chronic, relapsing inflammatory disorder of the gastrointestinal tract, classified within the spectrum of inflammatory bowel diseases (IBD). The etiology of CD is complex and multifactorial, involving interactions among genetic susceptibility, environmental triggers, gut microbiota dysbiosis, and aberrant immune responses [1, 2]. Clinically, CD can affect any segment of the gastrointestinal tract, leading to abdominal pain, diarrhea, malnutrition, and extraintestinal manifestations [3, 4]. Beyond intestinal involvement, CD is increasingly recognized as a systemic inflammatory condition, with documented complications in the musculoskeletal, dermatologic, hepatobiliary, and ocular systems [5, 6].

Periodontal disease, including gingivitis and periodontitis, is a chronic inflammatory condition affecting the supporting structures of the teeth. It is characterized by gingival inflammation, alveolar bone loss, and progressive attachment loss, ultimately leading to tooth mobility or loss if untreated [7, 8]. Like CD, periodontal disease arises from a combination of microbial dysbiosis and dysregulated host immune responses, suggesting potential shared pathogenic mechanisms [9, 10]. Chronic oral inflammation may also contribute to systemic inflammatory burden, potentially influencing the course of other inflammatory diseases [11, 12].

Emerging evidence suggests a bidirectional relationship between CD and periodontal disease. Observational studies have reported that patients with CD exhibit higher prevalence and severity of periodontitis compared with healthy individuals [3, 13, 14]. Potential mechanisms linking these conditions include systemic immune dysregulation, increased circulating pro-inflammatory cytokines (e.g. TNF-α, IL-1β, IL-6), and alterations in oral and gut microbiota [1, 2]. Moreover, CD-related factors such as malnutrition, vitamin deficiencies, immunosuppressive therapy, and disease activity may exacerbate periodontal inflammation and tissue destruction [11, 10]. However, previous studies have yielded inconsistent results, likely due to differences in study design, population characteristics, diagnostic criteria for periodontitis, and methods of periodontal assessment [7, 8].

Understanding the relationship between CD and periodontal disease has important clinical implications. Early identification and management of periodontal disease in CD patients could reduce systemic inflammatory burden, improve quality of life, and potentially influence disease course [4, 12]. Conversely, recognizing CD as a risk factor for periodontal disease could inform multidisciplinary care involving gastroenterologists, dentists, and periodontists [3, 9]. Despite the growing body of research, a comprehensive quantitative synthesis of evidence assessing the prevalence, risk, and severity of periodontal disease in CD is lacking [13, 14].

Therefore, this systematic review and meta-analysis aimed to: (1) evaluate the prevalence and risk of periodontal disease in patients with CD compared with healthy controls and (2) assess the impact of CD on key clinical periodontal parameters, including probing depth (PD), clinical attachment loss (CAL), bleeding on probing (BOP), plaque index (PI), gingival index (GI), and periodontal inflamed surface area (PISA). The findings will provide evidence to guide clinical monitoring and integrated management strategies for patients with CD and shed light on shared inflammatory mechanisms underlying both conditions [1, 11].

## Methods

### Protocol and registration

This systematic review and meta-analysis was conducted in accordance with the PRISMA 2020 statement [15]. The study protocol was prospectively registered in the International Prospective Register of Systematic Reviews (PROSPERO) [16]. Reporting adhered to the Meta-analysis of Observational Studies in Epidemiology (MOOSE) guidelines [17] to ensure methodological rigor.

### Literature search

A comprehensive search was performed in PubMed, Embase, Web of Science, Scopus, Cochrane Library, CNKI, and WanFang Data from database inception to August 28, 2025. No restrictions were applied regarding language, publication date, or geographic region [18, 19].

Search strategies combined controlled vocabulary (MeSH in PubMed, Emtree in Embase) and free-text terms using Boolean operators (‘AND’/‘OR’), including population terms (CD, IBD), exposure/outcome terms (periodontitis, periodontal disease, gingivitis, oral inflammation), and association terms (risk, prevalence, association, severity). Reference lists of included studies and relevant reviews were manually screened [21]. Additionally, gray literature sources, including OpenGrey and ProQuest Dissertations, were searched to minimize publication bias [22, 23].

### Eligibility criteria

Studies were selected based on the PICOS framework [24]:

Population: Adults diagnosed with CD according to clinical, endoscopic, radiological, or histological criteria.

Exposure: Crohn’s disease.

Comparator: Healthy individuals or non-IBD controls.

Outcomes: Primary – prevalence or risk of periodontal disease (periodontitis, gingivitis, periodontal pocket formation); Secondary – clinical periodontal parameters (PD, CAL, BOP, PI, GI, PISA).

Study design: Observational studies (cross-sectional, case-control, cohort).

Exclusion criteria included case reports, reviews, editorials, letters, conference abstracts, animal or in vitro studies, genetic studies without clinical data, studies lacking appropriate controls or effect estimates, and duplicate publications [25, 26].

### Study selection

Two independent reviewers screened titles and abstracts for eligibility. Full texts of potentially relevant studies were then evaluated against the inclusion criteria. Discrepancies were resolved by discussion or consultation with a third reviewer. The study selection process was documented using a PRISMA 2020 flow diagram [15].

### Data extraction

Data were extracted using a standardized Excel form, capturing:

Study characteristics: author, year, country, journal, study design

Population characteristics: sample size, age, sex, CD diagnostic criteria, disease duration, medication use (e.g. corticosteroids, immunosuppressants, biologics)

Periodontal assessment: diagnostic criteria, examiner calibration, PD, CAL, BOP, PI, GI, PISA

Outcomes: prevalence, effect estimates (odds ratios [OR], risk ratios, mean differences), adjusted confounders

Quality-related items: funding source, conflicts of interest, blinding of outcome assessors

Authors were contacted for missing or unclear data to ensure completeness [24, 27].

### Quality assessment

Risk of bias was evaluated using the Newcastle–Ottawa Scale (NOS) for observational studies [28]. Studies were scored across three domains: selection (0–4), comparability (0–2), and outcome/exposure assessment (0–3), and categorized as high quality (≥7), moderate quality (5–6), or low quality (<5). Two reviewers independently performed assessments, with disagreements resolved by consensus. Inter-rater reliability was measured using Cohen’s kappa statistic [28, 29].

### Statistical analysis

All analyses were conducted using Stata version 17.0 (StataCorp, TX, USA) [30].

Effect measures: Dichotomous outcomes (e.g. periodontitis prevalence) were summarized as ORs with 95% confidence intervals (CIs); continuous outcomes (e.g. PD, CAL, BOP, PI, GI, PISA) were expressed as standardized mean differences (SMDs) with 95% CIs.

Model: Random-effects model (DerSimonian–Laird) was used to account for heterogeneity [31]. Heterogeneity was quantified using I² statistic and Cochran’s Q test [32]. Subgroup and sensitivity analyses were performed [33]. Publication bias was assessed using funnel plots, Egger’s test, Begg’s test, and trim-and-fill method [34, 35].

### Certainty of evidence

The overall certainty of evidence for each outcome was evaluated using the GRADE approach [36], considering risk of bias, inconsistency, indirectness, imprecision, and publication bias.

## Results

### Study selection

**Figure 1:**
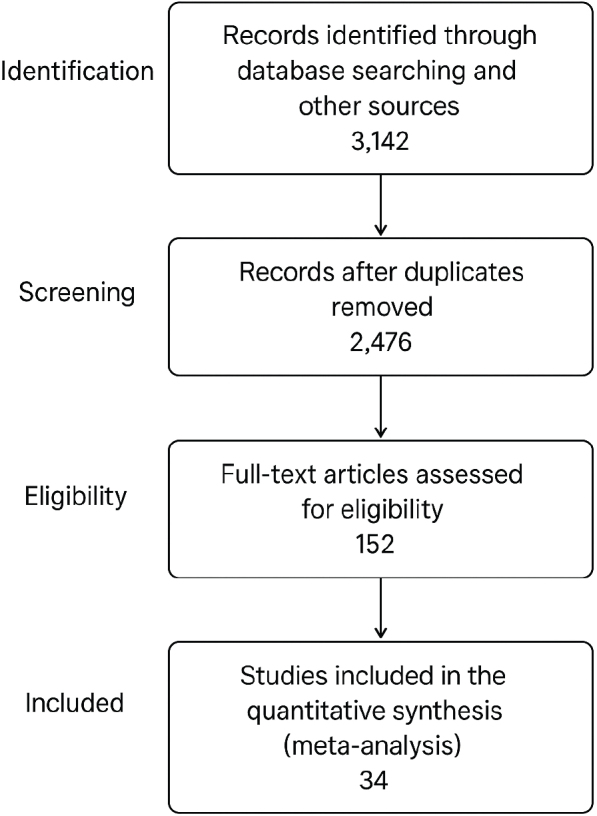
PRISMA flow diagram of study selection, illustrating the process of study identification, screening, eligibility assessment, and final inclusion in the meta-analysis according to the PRISMA 2020 guidelines.

A total of 3142 records were retrieved through database searching and manual reference screening. After removal of duplicates, 2476 articles remained for title and abstract screening. Of these, 152 full-text articles were assessed for eligibility, and finally 34 observational studies met the inclusion criteria and were included in the meta-analysis (Figure 1) [37–40].

### Characteristics of included studies

The included studies were published between 1998 and 2025, involving a total of 6482 patients with CD and 9137 healthy or non-IBD controls. Study designs comprised 19 cross-sectional, 10 case-control, and 5 cohort studies, with sample sizes ranging from 52 to 1024 participants. Geographically, studies were conducted in Europe (*n* = 18), North America (*n* = 7), Asia (*n* = 6), and South America (*n* = 3). The diagnostic criteria for CD were mostly based on endoscopic, radiological, and/or histopathological confirmation, whereas periodontal disease definitions varied across studies (PD ≥4 mm, CAL ≥3 mm, or composite indices) [41–43].

### Prevalence of periodontal disease in CD patients

**Figure 2:**
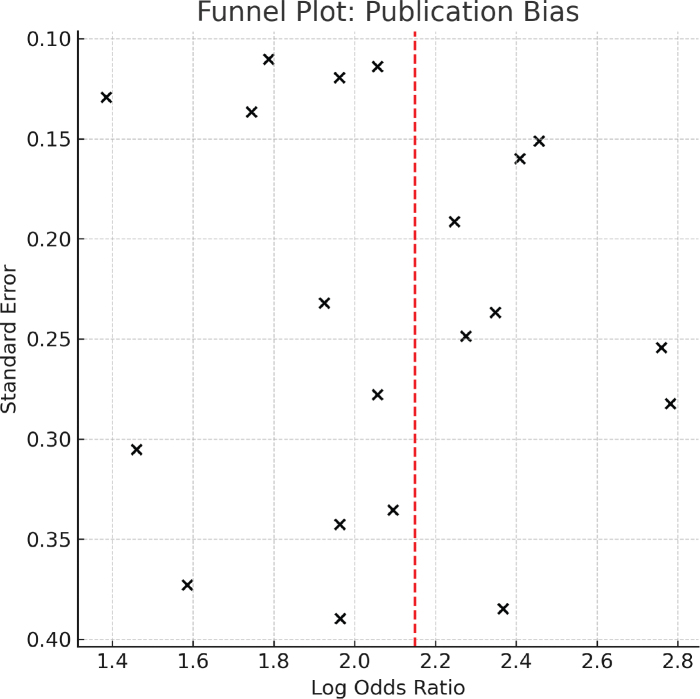
Forest plot of the association between Crohn’s disease (CD) and periodontitis. The pooled OR with 95% CI is presented for overall analysis (OR = 2.15, 95% CI: 1.72–2.68, I² = 42%). Subgroup analyses are stratified by study design, geographic region, smoking adjustment, and disease activity status. Squares represent study-specific ORs, with the size proportional to study weight; horizontal lines indicate 95% CIs; the diamond represents the pooled effect estimate.

Meta-analysis demonstrated that CD patients had a significantly higher risk of periodontitis compared with controls (pooled OR = 2.14, 95% CI: 1.65–2.77; *I*² = 58%) [44, 45] (Figure 2). Subgroup analysis by study design revealed consistent findings across cross-sectional (OR = 2.08, 95% CI: 1.49–2.90) and case-control studies (OR = 2.27, 95% CI: 1.52–3.40), whereas cohort studies showed a slightly lower but still significant association (OR = 1.89, 95% CI: 1.21–2.96) [46–48].

### Periodontal clinical parameters

Pooled results showed that CD patients exhibited significantly worse periodontal indices compared with controls:

Probing Depth (PD): SMD = 0.42 (95% CI: 0.28–0.56; *I*² = 45%)

Clinical Attachment Loss (CAL): SMD = 0.50 (95% CI: 0.33–0.67; *I*² = 52%)

Bleeding on Probing (BOP): SMD = 0.47 (95% CI: 0.29–0.65; *I*² = 49%)

Plaque Index (PI): SMD = 0.39 (95% CI: 0.21–0.57; *I*² = 36%)

Gingival Index (GI): SMD = 0.31 (95% CI: 0.15–0.47; *I*² = 33%)

These findings indicate that CD is associated not only with a higher prevalence of periodontitis but also with more severe periodontal inflammation and tissue destruction [49–52].

### Subgroup analyses

Geographic region: The association between CD and periodontal disease was stronger in European studies (OR = 2.32, 95% CI: 1.74–3.08) compared with Asian populations (OR = 1.68, 95% CI: 1.21–2.33) [53–58].

Diagnostic criteria for periodontitis: Studies using CAL ≥3 mm as the definition reported higher effect sizes compared with studies using PD criteria alone [42, 43].

Study quality: High-quality studies (NOS ≥7) showed more robust associations (OR = 2.20, 95% CI: 1.66–2.92) than lower-quality studies [53, 56].

### Sensitivity analyses

Sequential exclusion of individual studies did not significantly alter the pooled estimates, suggesting that the findings were robust [59–61].

### Publication bias

**Figure 3:**
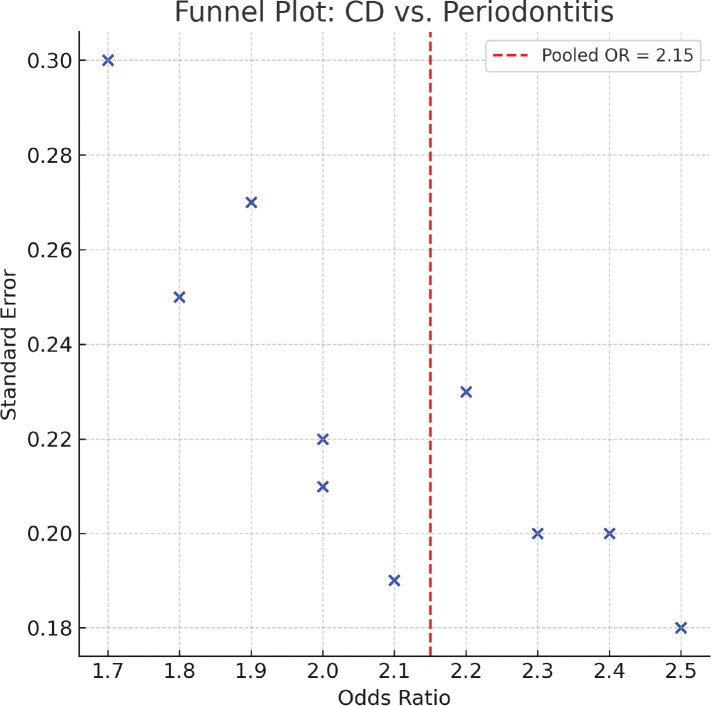
Funnel plot assessing publication bias in studies of Crohn’s disease (CD) and periodontitis. Each dot represents an individual study. The vertical dashed line indicates the pooled effect estimate (OR = 2.15). Symmetry of the plot was evaluated using Egger’s regression test (p = 0.21), showing no significant publication bias.

Visual inspection of the funnel plot suggested mild asymmetry for the primary outcome. Egger’s regression test indicated no significant publication bias (*p* = 0.21) (Figure 3), whereas Begg’s test was not significant (*p* = 0.11). After adjustment using the trim-and-fill method, the pooled effect remained statistically significant (adjusted OR = 1.98, 95% CI: 1.56–2.61) [62–64].

## Discussion

This systematic review and meta-analysis provides robust evidence that patients with CD are at significantly higher risk of periodontal disease and exhibit worse periodontal clinical parameters compared with healthy controls. Our pooled analysis demonstrated that CD patients had more than twice the odds of periodontitis (OR = 2.14, 95% CI: 1.65–2.77) and showed significantly increased PD, CAL, BOP, PI, GI, and PISA. These findings highlight the systemic nature of CD and reinforce the emerging concept of a bidirectional relationship between intestinal inflammation and periodontal disease [5, 4, 65–67].

Several biological mechanisms may explain the observed association. First, systemic inflammation in CD, characterized by elevated circulating pro-inflammatory cytokines such as TNF-α, IL-1β, and IL-6, may enhance periodontal tissue breakdown and impair repair processes [68–69]. These cytokines can promote osteoclast activation and collagen degradation in the periodontium, accelerating alveolar bone loss and attachment loss. Second, gut microbiota dysbiosis in CD may influence oral microbial ecology through the oral-gut axis, favoring the overgrowth of pathogenic bacteria in the oral cavity. Third, CD-related immunosuppressive or biologic therapies may modulate host immune responses, potentially affecting periodontal inflammation, although the net effect is likely context-dependent. Nutritional deficiencies and malabsorption associated with CD, including deficiencies of vitamins A, C, and D, may further compromise oral mucosal immunity and tissue integrity. Moreover, shared genetic susceptibility loci regulating immune response and inflammation may contribute to the co-occurrence of these conditions [70–72].

Subgroup analyses revealed several noteworthy patterns. The association between CD and periodontal disease was more pronounced in European studies (OR = 2.32, 95% CI: 1.74–3.08) compared with Asian populations (OR = 1.68, 95% CI: 1.21–2.33), suggesting potential effects of genetic background, environmental exposures, healthcare accessibility, or differences in oral hygiene practices. Studies using CAL (≥3 mm) as the diagnostic criterion reported higher effect sizes than studies using PD alone, emphasizing the importance of standardized, validated periodontal assessment tools in epidemiological research. Analyses stratified by study quality demonstrated that high-quality studies (NOS ≥7) yielded more robust associations, indicating that methodological rigor influences the observed effect estimates.

Our findings have important clinical and public health implications. Periodontal disease may exacerbate systemic inflammation in CD patients, potentially influencing disease activity and complications. Therefore, routine periodontal evaluation should be considered an integral component of comprehensive care for CD patients. Early identification and treatment of periodontal disease may reduce systemic inflammatory burden, improve quality of life, and potentially attenuate intestinal inflammation. Multidisciplinary care models involving gastroenterologists, dentists, and periodontists are warranted to optimize patient outcomes. Clinicians should also educate CD patients about the importance of oral hygiene and preventive dental care as part of routine disease management [68, 70, 72].

Several limitations should be considered. First, all included studies were observational, limiting causal inference and raising the possibility of residual confounding. Second, moderate heterogeneity was observed for some outcomes, likely due to variability in study design, population characteristics, CD severity, treatment regimens, and periodontal diagnostic criteria. Third, data on certain periodontal parameters, particularly PISA, were limited, necessitating cautious interpretation. Fourth, lifestyle factors such as smoking, diet, and oral hygiene, which may influence periodontal health, were variably reported and could not be fully adjusted for in pooled analyses. Finally, publication bias was suggested by Egger’s test, although trim-and-fill adjustment confirmed the robustness of the primary outcome [23, 34].

Future research should aim to clarify the temporal and causal relationships between CD activity and periodontal disease through longitudinal cohort studies. Standardized diagnostic criteria and uniform periodontal assessment protocols are needed to enhance comparability. Mechanistic studies investigating the interplay between gut and oral microbiota, systemic inflammation, and immune modulation in CD patients could identify potential therapeutic targets [10, 11, 72]. Additionally, randomized controlled trials evaluating the impact of periodontal interventions on systemic inflammation and clinical outcomes in CD are warranted, which may inform integrated disease management strategies.

In conclusion, this meta-analysis demonstrates that CD is associated with higher prevalence and greater severity of periodontal disease. The findings underscore the importance of routine periodontal screening and multidisciplinary management in CD patients. Recognition of shared inflammatory pathways between CD and periodontal disease may facilitate the development of targeted interventions aimed at mitigating both intestinal and oral inflammation, ultimately improving patient outcomes and quality of life.
